# Cardiac amyloidosis: diagnostic challenges and recent advancement in the treatment of transthyretin amyloidosis (ATTR)

**DOI:** 10.1093/omcr/omab059

**Published:** 2021-08-13

**Authors:** Tanvir Rahman, Reihaneh C Moghadam, Vikram V Agarwal, Craig K Reiss

**Affiliations:** 1 Department of Internal Medicine, St. Luke’s Hospital, Chesterfield, MO, USA; 2 Department of Cardiology, St. Luke’s Hospital, Chesterfield, MO, USA

## Abstract

ATTR-CA is an under-reported cause of congestive heart failure (CHF) and cardiac arrhythmias. Heightened clinical suspicion along with a multimodal investigative approach is often required in diagnosing this potentially fatal condition. Tafamidis and inotersen have shown promising results in terms of progression-free survival by ameliorating CHF symptoms and peripheral neuropathies in clinical trials.

In this case series of five patients, we present three wild-type cardiac amyloidosis (ATTRwt-CA), one familial cardiac amyloidosis (ATTRm-CA) and one primary cardiac (AL-CA). The diagnostic modality was different for each patient. ATTRwt-CA, ATTRm-CA and AL-CA patients received tafamidis, inotersen and chemotherapy with bone marrow stem-cell transplantation, respectively.

## INTRODUCTION

Amyloidosis is caused by the deposition of misfolded insoluble beta-pleated sheets into the extracellular space. Transthyretin cardiac amyloidosis (ATTR-CA) could be present in a substantial number of patients with congestive heart failure (CHF) and new-onset dyspnea and is often overlooked. Wild-type ATTR (ATTRwt-CA) accounts for up to 13% of heart failure with preserved ejection fraction cases. The common clinical manifestations of ATTRwt-CA are related to CHF including dyspnea on exertion (DOE), orthopnea, paroxysmal nocturnal dyspnea, elevated jugular venous pressure, pulmonary crackles and edema [1]. The presence of a low-flow, low-gradient aortic stenosis phenotype in individuals with aortic stenosis is also reported to be associated with ATTRwt-CA [2]. Electrocardiogram (EKG) findings suggestive of ATTR-CA include low QRS voltage (LQRSV) [3] and conduction abnormalities [4]. Associated orthopedic manifestations including bilateral carpal tunnel syndrome (CTS) [5], lumbar spinal stenosis [6] and biceps tendon rupture [7]. No single definitive test is available to diagnose ATTR-CA and a multimodal approach is often required. We diagnosed five cases of ATTR-CA in one physician’s practice within 1 year. In this case series, we aim to discuss the diagnostic challenges encountered with different types of ATTR-CA. We also highlight advances in the treatment of ATTR-CA and patients receiving novel therapies.

**Figure 1:**
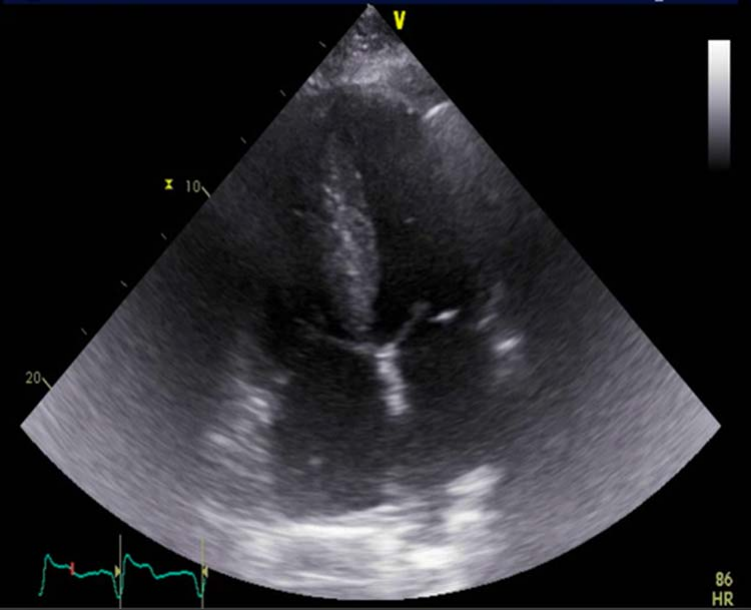
A TTE showing homogenous speckled myocardium.

**Figure 2:**
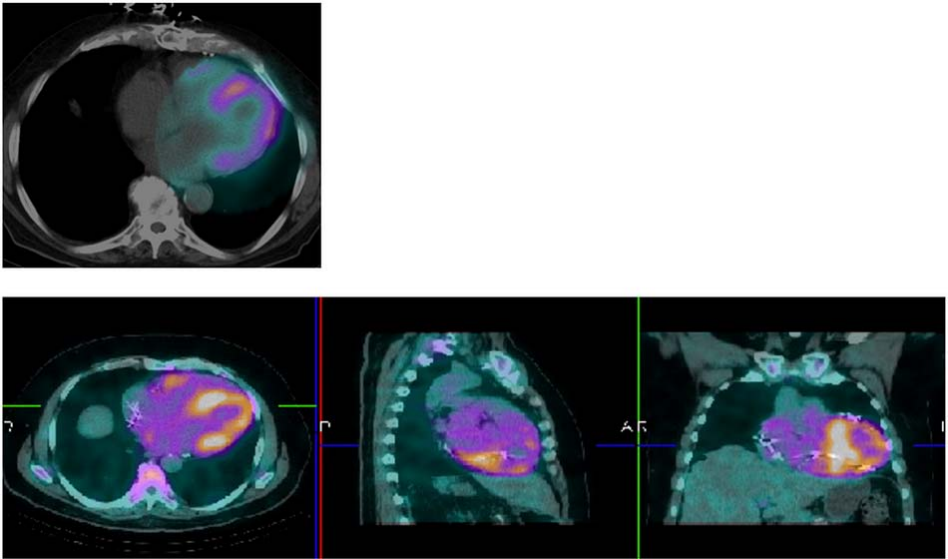
Perugini grade 3 uptake by myocardium consistent with TTR amyloidosis.

## CASE REPORT 1

A 71-year-old male presented with DOE and syncopal episodes. His past medical history (PMH) was notable for hypothyroidism, hypertension (HTN), type II diabetes mellitus, obstructive sleep apnea (OSA), CTS, CHF, stage IV chronic kidney disease (CKD IV), complete heart block post-successful permanent pacemaker, paroxysmal atrial fibrillation (AFib). Labs were significant for mildly elevated troponin I (TnI) 0.12 ng/ml, elevated NT-proBNP 9655 pg/ml. EKG showed no acute ST-T changes. Transthoracic echocardiogram (TTE) was suggestive of infiltrative cardiomyopathy (Fig. 1).

Tc-99 m pyrophosphate (PYP) single-photon-emission computed tomography demonstrated diffuse uptake of PYP throughout the left ventricle myocardium and increased uptake in the right ventricle free wall (Perugini Grade 3), consistent with ATTR-CA (Fig. 2). A clinical diagnosis was made and myocardial biopsy was not recommended. Bone marrow aspiration and biopsy (BMBA) was negative for multiple myeloma (MM). Left heart catheterization was nonrevealing and saliva genetic test for ATTR was negative for any mutation.

In addition to the CHF management, the patient was started on Tafamidis 80 mg daily for ATTRwt-CA. He received an implantable cardioverter-defibrillator for low ejection fraction (EF). AFib was treated with amiodarone and apixaban. The patient progressed to CKD IV. CHF worsened and required multiple admissions with two abdominal paracenteses. NT-proBNP worsened to 31 900 pg/ml. EKG was notable for LQRSV. He developed acute encephalopathy from end-stage renal disease, cardiorenal syndrome and unfortunately passed away.

## CASE REPORT 2

**Figure 3:**
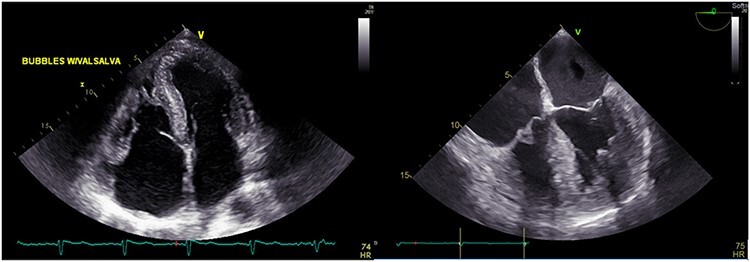
TTE showing speckled myocardium suggesting infiltrative cardiomyopathy, moderate concentric left ventricular hypertrophy (LVH) and bi-atrial enlargement.

**Figure 4:**
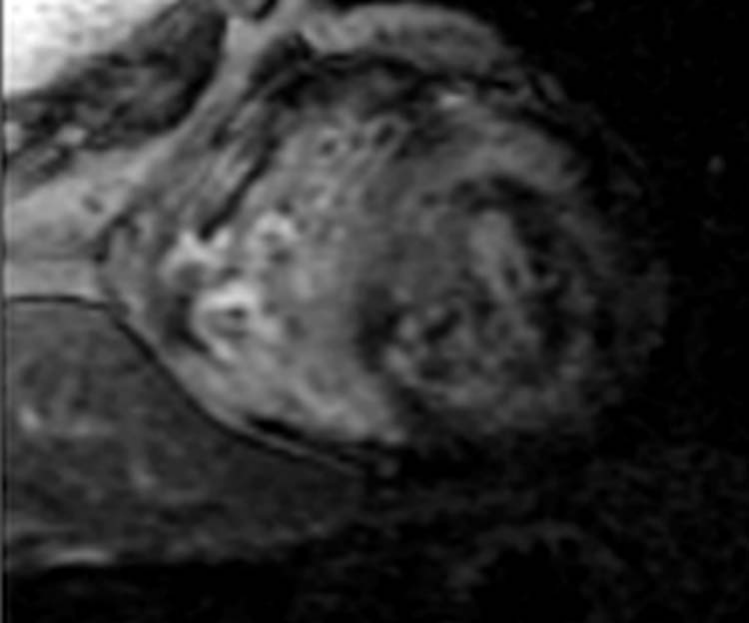
CMR showing concentric left ventricular hypertrophy.

**Figure 5:**
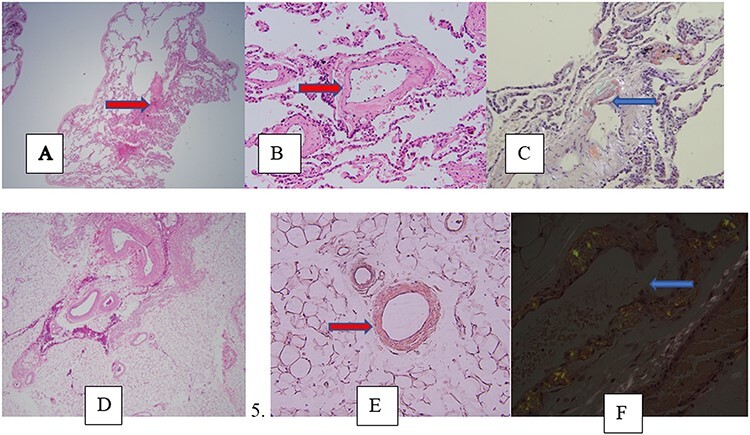
imaging and pathology of ATTR-CA A-C (Patient 4). (**A**) Low-power view ×100, lung tissue with thickened eosinophilic vessels (hematoxylin and eosin [H&E] stain); (B) high- power ×200 view of the same slide; (**C**) high power ×400 view Congo-red stain showing apple-green birefringence under polarized light. (**D**–**F**) (Patient 2). (D) ×100 view abdominal fat pad biopsy showing thickened eosinophilic blood vessels (H&E stain); (E) ×200 view on Congo-red staining without polarized light. (F) High-power ×400 view Congo-red stain showing apple-green birefringence under polarized light.

A 75-year-old male presented with progressive DOE for the past 1 ½ years. The patient used to be an avid runner. His PMH was notable for scleroderma, OSA and paroxysmal AFib. Investigations reported negative TnI, elevated NT-proBNP at 2880 pg/ml and LQRSV. TTE was suggestive of infiltrative cardiomyopathy (Fig. 3). Cardiovascular magnetic resonance imaging (CMR) was suggestive of infiltrative cardiomyopathy (Fig. 4). The positron emission tomography scan was nonrevealing. BMBA showed increased plasma cells. Myeloma fluorescence *in situ* hybridization was positive for translocation 11;14 and monosomy 13 and negative for TP53 deletion. Abdominal fat pad biopsy was positive for amyloidosis (Fig. 5).

The patient received chemotherapy with bortezomib, lenalidomide and dexamethasone (VRD regimen) and subsequent hematopoietic stem cell transplantation (HSCT) in addition to the CHF and AFib management. The patient did remarkably well after the HSCT with a significant decrease in light chains and exercise tolerance improved.

## CASE REPORT 3

**Figure 6:**
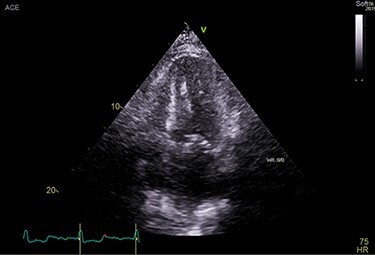
TTE showing increased echogenicity in the ventricular septum.

A 73-year-old African-American female presented with worsening DOE, peripheral edema, worsening peripheral neuropathy (PN). Her PMH was notable for type-1 diabetes mellitus, hypothyroidism, OSA, HTN, hyperlipidemia (HLD), coronary artery disease status post coronary artery bypass grafting (CAD S/P CABG), PN, CTS. Her sister passed away from ATTR-CA. TTE was suggestive of infiltrative cardiomyopathy (Fig. 6). CMR and Tc99-PYP scan were nondiagnostic. Genetic testing for saliva was positive for pathogenic mutation PV 142I heterozygous, suggestive of ATTRm-CA.

The patient was started on inotersen in addition to the CHF management. She tolerated inotersen well except for mild thrombocytopenia. Her DOE and peripheral edema had significantly improved in follow-up (F/U) visits. Her daughter tested positive for ATTRm-CA as well.

## CASE REPORT 4

**Figure 7:**
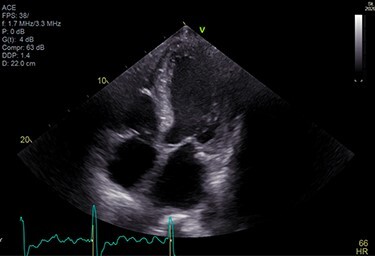
TTE showing moderate concentric LVH, severe left atrial enlargement.

**Figure 8:**
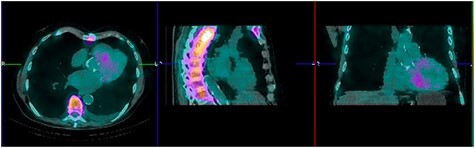
Technetium PYP scan was equivocal with patchy PYP uptake.

An 83-year-old white male presented with nonexertional chest discomfort, easy bruising and bleeding. His PMH was notable for HLD, OSA, CKD-IV, permanent AFib, sick sinus syndrome, CHF, CAD s/p CABG. Mildly elevated TnI at 0.10 ng/ml and elevated NT-proBNP at 10700 pg/ml was noted. EKG showed AFib and LQRSV. TTE resembled infiltrative cardiomyopathy (Fig. 7). Holter monitor showed a significant premature ventricular contraction (PVC) burden (>24 000 PVC’s/day, or 41%). The nuclear stress test, Tc99-PYP and genetic analysis were nonrevealing (Fig. 8). Left lung upper lobe wedge biopsy was positive for amyloidosis (Fig. 5).

The patient was started on tafamidis. PVCs ablated, CHF and AFib were adequately treated. The patient tolerated the tafamidis well with an improved shortness of breath (SOB) in F/U visits.

## CASE REPORT 5

**Figure 9:**
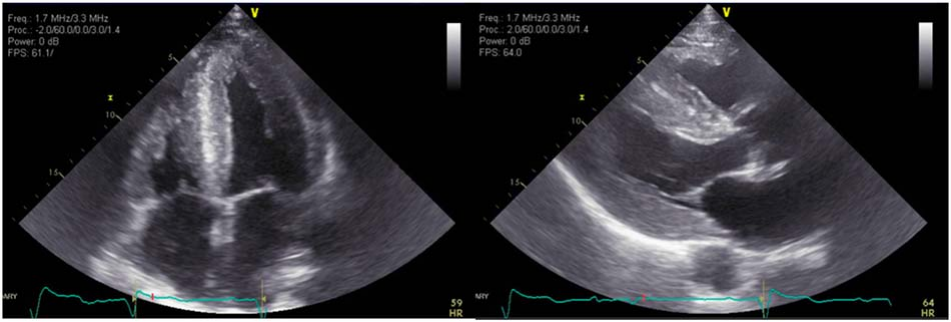
TTE showing increased myocardial echogenicity suggestive of ATTR-CA.

A 70-year-old African-American male, football coach presented with DOE. His PMH was notable for HTN, HLD, OSA, CAD s/p percutaneous coronary intervention (PCI), PN, CTS, permanent AFib. His lab results reported elevated NT-proBNP at 2369 pg/ml. TTE was suggestive of infiltrative cardiomyopathy (Fig. 9).

CMR showed late gadolinium enhancement in all four chambers, compatible with infiltrative cardiomyopathy. MM workup was negative. Endomyocardial biopsy was positive for amyloidosis. In addition, genetic testing for saliva was positive for ATTR. The patient was started on tafamidis for ATTRwt-CA in addition to the treatment of CHF and AF. His SOB and chest discomfort improved at the 3-month F/U visit.

The five cases are summarized in Table 1.

**Table 1:** Summary of the patients

Patient no.	Major EKG changes^*^	TTE	Diagnosis confirmation	Type of amyloidosis	Treatment
1	AFib	Severe LVH, Grade III Diastolic Dysfunction (DD), Left Ventricular Ejection Fraction (LVEF) 35–40%,	Tc99-PYP scan: Perugini grade 3 uptake	ATTRwt-CA	Tafamidis
2	AFib, Right Bundle Branch Block (RBBB)	Moderate LVH, severe Left Atrial Enlargement (LAE), LVEF 60%	CM: late gadolinium enhancement (LGE) Biopsy: abdominal fat pad, bone marrow	AL-CA	VRD regimen plus HSCT
3	High-degree Atrioventricular (AV) block, LQRSV in precordial leads	Moderate LVH, Grade II DD, LVEF 64%, increased ventricular septal echogenicity	Genetic test: saliva, pathogenic mutation PV 142I, heterozygous	ATTRm-CA	Inotersen
4	AFib with rapid ventricular response (RVR), prolonged QT	Moderate LVH, severe LAE, LVEF 38%	Biopsy: left upper lobe wedge biopsy of the lung	ATTRwt-CA	Tafamidis
5	AFib, variable AV block, RBBB	Marked LVH, Grade III DD, LVEF 55%, moderately dilated Left Atrium (LA), increased myocardial echogenicity	CMR: diffuse subendocardial delayed contrast enhancement involving all four chambers Biopsy: endomyocardial Genetic test: saliva	ATTRwt-CA	Tafamidis

^*^All patients had age indeterminate ST-T wave abnormality/ischemic changes

## DISCUSSION

The ‘gold standard’ test for diagnosis of ATTR-CA is a myocardial biopsy, which is an invasive procedure. Thus, in practice, the diagnosis of cardiac amyloidosis is usually made by echocardiography, supported by a noncardiac biopsy. In this case series diagnosis of cardiac amyloidosis required multiple diagnostic modalities including TTE, Tc99-PYP scan, CMR, saliva genetic testing and biopsy.

Treatment of ATTR-CA varies with its chemical composition. AL-CA is treated with chemotherapy and HSCT [8]. There have been recent advances in the treatment of ATTRwt-CA and ATTRm-CA including transthyretin stabilizers inhibiting the breakdown of transthyretin tetramer to monomer, disrupting the formation of amyloid fibril. Food and Drug Administration has approved tafamidis (ATTR-ACT trial), which showed lower all-cause mortality and inpatient admissions with cardiovascular complications compared with placebo several years post-initiation [9]. Patisiran (APOLLO trial) is a small interfering RNA and inotersen (NEURO-TTR trial) is an antisense oligonucleotide both of which degrade the mRNA of TTR inhibiting the production of transthyretin in the liver [10]. Investigational drugs like AG10 and vutrisiran are undergoing clinical trials [9].

In this case series, three cases of ATTRwt-CA receiving tafamidis, one case of ATTRm-CA receiving inotersen and one case of AL-CA receiving VRD regimen plus HSCT were presented. All of the patients responded well to the treatment and were symptom free in the F/U visits.

In summary, ATTR-CA is an underreported cause of CHF and cardiac arrhythmias. Heightened clinical suspicion along with a multimodal investigative approach is often required in diagnosing this potentially fatal condition. Tafamidis and inotersen have shown promising results in terms of progression-free survival by ameliorating CHF symptoms and PNs in clinical trials.
